# Diversity and ecological niche model of malaria vector and non-vector mosquito species in Covè, Ouinhi, and Zangnanado, Southern Benin

**DOI:** 10.1038/s41598-024-67919-5

**Published:** 2024-07-23

**Authors:** Constantin Jésukèdè Adoha, Arthur Sovi, Germain Gil Padonou, Boulais Yovogan, Bruno Akinro, Manfred Accrombessi, Edouard Dangbénon, Aboubakar Sidick, Razaki Ossè, Tachémè Filémon Tokponon, Esdras Mahoutin Odjo, Come Z. Koukpo, Arsène Fassinou, Antoine A. Missihoun, André Sominanhouin, Louisa A. Messenger, Prudenciène A. Agboho, Serge Akpodji, Corine Ngufor, Jackie Cook, Clément Agbangla, Natacha Protopopoff, Manisha A. Kulkarni, Martin C. Akogbéto

**Affiliations:** 1 Faculté des Sciences et Techniques, Université d’Abomey-Calavi, Abomey-Calavi, Benin; 2 Centre de Recherche Entomologique de Cotonou, Cotonou, Benin; 3 Faculty of Infectious and Tropical Diseases, Disease Control Department, London School of Hygiene and Tropical Medicine, London, WC1E 7HT UK; 4 Faculté d’Agronomie, Université de Parakou, Parakou, Benin; 5 Ecole de Gestion et d’Exploitation des Systèmes d’Elevage, Université Nationale d’Agriculture, Kétou, Benin; 6 Parasitology and Vector Biology Laboratory (UNLV PARAVEC Lab), School of Public Health, University of Nevada, Las Vegas, NV USA; 7 Department of Environmental and Occupational Health, School of Public Health, University of Nevada, Las Vegas, NV 89154 USA; 8 Medical Research Council (MRC) International Statistics and Epidemiology Group, London School of Hygiene and Tropical Medicine, London, WC1E 7HT UK; 9 School of Epidemiology and Public Health, University of Ottawa, Ottawa, ON Canada

**Keywords:** Diversity, Seasonal abundance, Ecological niche, Mosquito species, Disease vector, Entomology, Ecological modelling

## Abstract

The present study aimed to assess mosquito species diversity, distribution, and ecological preferences in the Covè, Ouinhi, and Zangnanado communes, Southern Benin. Such information is critical to understand mosquito bio-ecology and to focus control efforts in high-risk areas for vector-borne diseases. Mosquito collections occurred quarterly in 60 clusters between June 2020 and April 2021, using human landing catches. In addition to the seasonal mosquito abundance, Shannon's diversity, Simpson, and Pielou's equitability indices were also evaluated to assess mosquito diversity. Ecological niche models were developed with MaxEnt using environmental variables to assess species distribution. Overall, mosquito density was higher in the wet season than in the dry season in all communes. A significantly higher Shannon's diversity index was also observed in the wet season than in the dry seasons in all communes (p < 0.05). Habitat suitability of *An. gambiae* s.s.*, An. coluzzii, Cx. quinquefasciatus* and *Ma. africana* was highly influenced by slope, isothermality, site aspect, elevation, and precipitation seasonality in both wet and dry seasons. Overall, depending on the season, the ecological preferences of the four main mosquito species were variable across study communes. This emphasizes the impact of environmental conditions on mosquito species distribution. Moreover, mosquito populations were found to be more diverse in the wet season compared to the dry season.

## Introduction

Mosquitoes play a crucial role in the spread of multiple infectious diseases such as malaria, dengue, chikungunya, and yellow fever, whose epidemics can lead to severe health and economic consequences for populations^1–3^. This issue is particularly concerning in Benin, where the malaria incidence rate of 2021 was 383.4 cases per 1000 with about 11,154 deaths^4^. In addition, the true epidemiological burden of the other vector-borne diseases is still unknown due to a dearth of data.

The spread of vector-borne diseases is intrinsically linked to the environment, which provides conducive conditions for the emergence and proliferation of vectors^5,6^. Given the pivotal role played by mosquitoes in the transmission of infectious pathogens, a comprehensive understanding of their bio-ecology is required to better assess health risks, and devise effective prevention and control strategies^7^. Thus, investigating and characterizing the mosquito fauna and their distribution becomes crucial. A study conducted in 2019 in the communes of Covè, Ouinhi and Zangnanado in Southern Benin showed the presence of mosquitoes of the *Anopheles*, *Aedes*, *Culex* and *Mansonia* genera^8^. However, with the emergence of invasive mosquito species such as *Anopheles stephensi* and *Aedes albopictus* in neighbouring countries such as Nigeria and Ghana^9,10^, an update of the local mosquito fauna, as well as the understanding of its geographical distribution, and explanatory environmental factors are required.

Species distribution models are likely the most suitable tools to aid in understanding the relationships between vectors and their environment^11^. These models provide an efficient approach to map the presence and abundance of mosquito species in a given area, thereby facilitating the identification of areas with potential risk or high risk of emergence of vector-borne diseases^12,13^. These maps provide critical information that will help focus efforts for controlling disease-transmitting mosquitoes in high risk areas. Moreover, these models enhance our understanding of the ecological and environmental factors influencing mosquito presence and their geographical distribution^11,12,14^. These factors include temperature, humidity, precipitation, vegetation cover, and proximity to water bodies^14^.

The present study aimed to assess the mosquito species composition, their distribution, and ecological preferences in the communes of Covè, Zangnanado, and Ouinhi, three communes of Southern Benin with different ecological characteristics, and endemic vector-borne diseases such as malaria^15^ and lymphatic filariasis (LF)^16^.

## Materials and methods

### Study area

**Figure 1:**
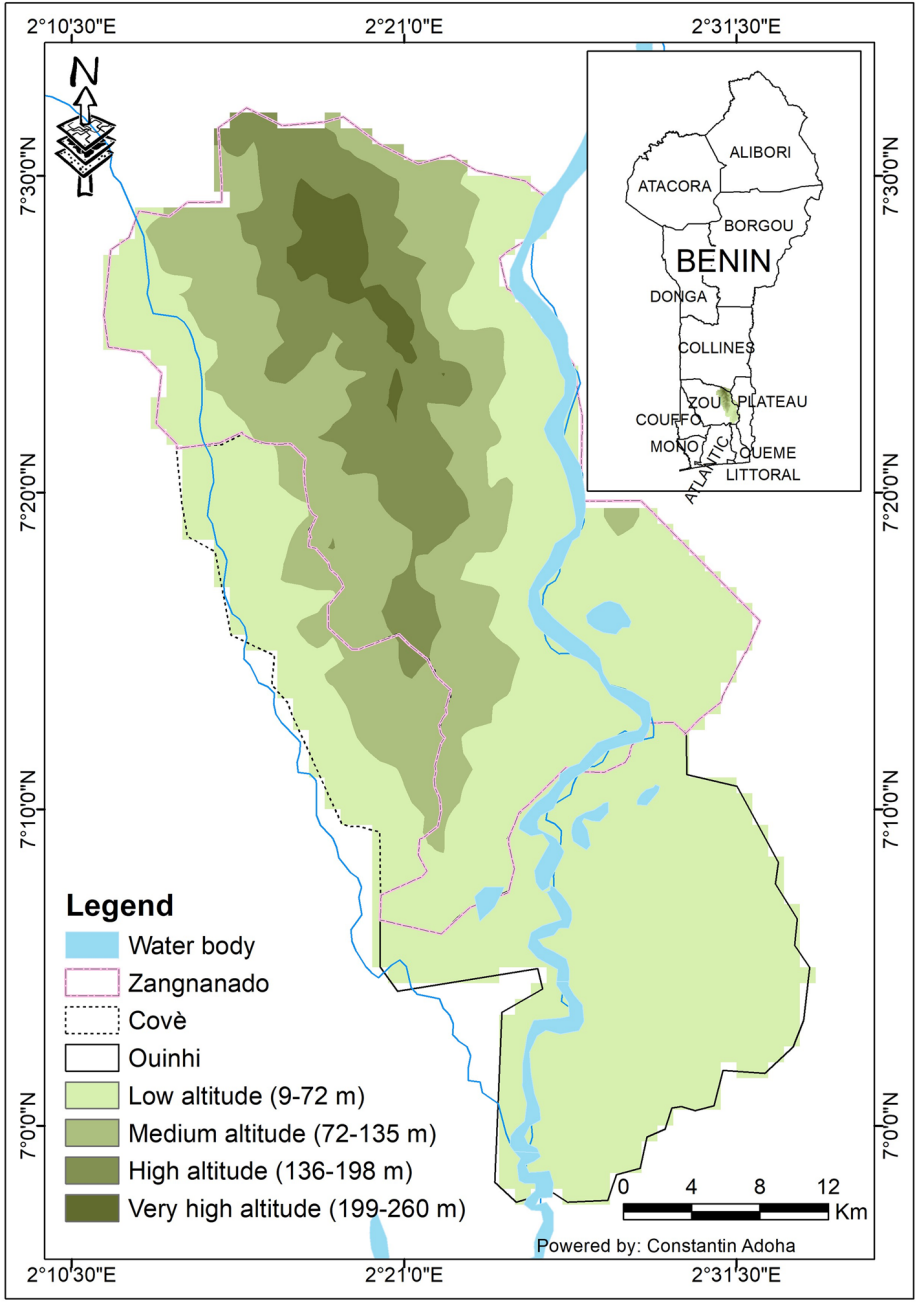
Map of the study area. The map was drawn by C.J.A. using ArcGIS Desktop 10.8.1 software, study and online data was provided by Noce et al*. *^22^.

The present study is a secondary analysis using data collected from a large randomised controlled trial (RCT) designed to assess the efficacy of new types of mosquito bed nets for the control of malaria. The main RCT was conducted in the communes of Covè (07°13′08.0400″ N, 02°20′21.8400″ E), Ouinhi (07°05′00″ N, 02°29′00″ E), and Zangnanado (07°16′00″ N, 02°21′00″ E) in the Zou department (Fig. 1), located 154 km north of Cotonou, the economic capital of Benin. Annual rainfall varied between 985 and 1200 mm and was the lowest in Zangnanado^17,18^. The malaria infection prevalence was 36.5% in under 5 years old children^15^. Elevation ranged between 9 to 250 m above sea level with the highest elevation in Zangnanado.

The study area covers 1758 km^2^, with a population of approximately 220,000 inhabitants, residing in around 54,000 households distributed across 123 villages^19,20^ grouped into 60 clusters^21^. The economic activities of the population include agriculture, fishing, hunting, and trade^19^. The area has a Sudano-Guinean climate.

### Collection, processing and occurrences of mosquito species

Collections of adult mosquitoes were carried out every three months across study clusters between June 2020 and April 2021. In each cluster, four households located 15–20 m apart were selected to ease the overnight supervision of mosquito collections that occurred using the human landing catches (HLC) technique. Four collectors were assigned to each household, with two collectors (one indoor and one outdoor) collecting mosquitoes from 19:00 to 01:00, and the second group collecting from 01:00 to 07:00 in the morning. Mosquito specimens collected through HLCs were morphologically identified and separated as Culicinae and Anophelinae using a binocular loupe. Anophelinae mosquitoes were identified at the species level using the keys of Gillies and De Meillon^23^ and Gillies and Coetzee^24^. Culicinae mosquitoes were also identified at the species level using the identification keys of Doby (1955) and Edwards (1941). Specimens of *An. gambiae* s.l. were preserved using silica gel and molecularly identified using the protocol of Santolamazza et al*.*^25^.

Two databases, one with data collected during the wet season (June to November 2020), and the second with data collected over the dry season (December 2020 to April 2021) were generated. Each database included five mosquito species, of which three (*An. gambiae* sensu lacto, *Cx. quinquefasciatus*, and *Ma. africana*) were morphologically identified, and two (*An. gambiae* s.s., *An. coluzzii*) were molecularly identified. The databases contained both occurrence and mosquito abundance data. The geographical coordinates of mosquito collection households were used as occurrence data points for the different mosquito species. Households where a species was absent were excluded from the occurrence data for that species. Species occurrence points were rarefied at a resolution of 500 m to avoid pseudo-replication of data points and reduce clustering.

### Climatic and environmental data

Overall, a total of 23 topographical, climatic and landscape variables were obtained to build the models (Supplementary Table S1). The 19 bioclimatic variables (bio1 to bio19), slope, Shuttle Radar Topography Mission (SRTM) elevation, soil type and aspect datasets were downloaded from the WorldClim database at a spatial resolution of 30 arc seconds (~ 1 km)^26^. Landsat 8 and 9 satellite data were downloaded from the USGS website^27^ and the normalized differential vegetation index (NDVI) was calculated using ArcGIS software. All raster layers were cropped to the extent of the Covè-Zangnanado-Ouinhi communes, resampled to 1000 m resolution using bilinear interpolation, and transformed to the projected WGS 1984 World Mercator coordinate system using the Extract by Mask function in ArcGIS 10.8. The correlation between bioclimatic variables was checked, and highly correlated variables (Pearson's correlation coefficients r > 0.7) were excluded from the model. To maximise model performance, minimise model overfitting (minimise the number of variables in the model) and multicollinearity (minimise the correlation between variables in the models), we used Pearson's correlation coefficient (r) to select the final predictor variables from the initial pool of 19 climate variables described above. Annual mean temperature (bio1), isothermality (bio3), maximum temperature of warmest month (bio5), annual precipitation (bio12), seasonality of precipitation (bio15), Precipitation of Wettest Quarter (bio16) were the less correlated bioclimatic variables (r < 0. 7) that were retained to build the ecological niche models for *An. gambiae* s.s., *An. coluzzii*, *Cx. quinquefasciatus* and *Ma. africana* during the dry and wet seasons.

### Ecological niche models

Due to its robust predictive accuracy^28^, predictive performance using presence-only data^29^ and insensitivity to sample size^30^, the maximum entropy (MaxEnt) modelling approach is frequently used to develop ecological niche models.

To generate habitat suitability maps for *An. gambiae* s.s., *An. coluzzii*, *Cx. quinquefasciatus* and *Ma. africana*, 12 topographic, landscape and climatic variables were applied as predictor environmental variables alongside species occurrence data using MaxEnt version 3.4.4^31^. The selected output option was “cloglog” which produces habitat suitability estimates in a range between 0 and 1 for each pixel. The modelling process for each species used k-fold cross-validation replicated over 20 iterations to partition the occurrence data and use all presence points for training and testing^32^. MaxEnt parameters were configured with a maximum of 10,000 iterations, keeping other parameters as default. Linear, quadratic and product transformations of the environmental variables were used with a regularisation multiplier of 1.

Several methods including the jackknife test and permutation techniques, were used to assess the importance of the variables in the model. The jackknife procedure was used to assess the regularized training gain of models built using each variable individually, and when each variable was excluded from the model in turn. Variables for which the model gain decreased the most when omitted or which increased the model gain the most when considered in isolation were identified as the most informative variables in the model that contain information not found in the other variables. Measuring importance by permutation involves randomly permuting the values of each variable between the presence and background data, and then re-evaluating the model. The resulting variation in fit performance is calculated for each variable, where a more significant variation indicates an increased importance of that variable.

Finally, the assessment of model performance for each species used the area under the receiver operating characteristics curve (AUC) based on the testing data, calculated as an average of the AUCs obtained over the 20 iterations of the model. An AUC of 1 indicates perfect prediction, while an AUC of 0.5 suggests prediction equivalent to chance.

### Data analysis

To scrutinize the structure of the mosquito population within each commune, three indicators were determined:

- The Shannon's diversity index (H′) assesses the different types of mosquito species present. It ranges between 0 (homogeneous population consisting of only one species) and Hʹmax equals to log S (heterogeneous population for which all individuals of all species are equally distributed), with S being the species richness (total number of species identified in a given area). This was calculated as follows:
  $${\text{H}}^{\prime} \, = \, - \sum \, \left( {{\text{ni}}/{\text{N}}} \right) * {\text{log }}\left( {{\text{ni}}/{\text{N}}} \right)$$
  where "ni" denotes the number of individuals belonging to a given species and "N" represents the overall number of individuals of all mosquito species in a commune.
- The Simpson index (1 − D) evaluates the predominance of a given species within the population. It ranges from 0 (lower diversity with relatively few dominant species in the population) to 1 (higher diversity with several equally distributed species in the population). The calculation of this index was done as follows:
  $${1} - {\text{D }} = { 1} - \sum {\text{ ni }}\left( {{\text{ni}} - {1}} \right)/{\text{ N }}\left( {{\text{N}} - {1}} \right)$$
  where “ni” is the number of individuals of the given species and “N” is the total number of individuals.
- The Pielou's equitability index (Jʹ) measures the evenness of species distribution in a population and derives from the Shannon diversity index (Hʹ). The Pielou's equitability index ranges from 0 (minimum evenness, suggesting a highly skewed distribution where one or a few species dominate the population) to 1 (maximum evenness which means that all species in the population have equal abundance). The formula to calculate this index is the following:
  $${\text{J}}^{\prime} = {\text{ H}}^{\prime} /{\text{H}}^{\prime} {\text{max}}$$
  where "Hʹ" is Shannon's diversity index and "Hʹmax" is the maximum possible value of Hʹ if each species has the same probability.

The diversity indices were calculated using Past version 3.14 software^33^. They were compared between communes using the Tukey test (package Agricolae). This test was performed using R software version 4.3.0, with the statistical significance threshold set at 0.05.

The mean density of mosquitoes per season was calculated for *An. gambiae* s.l, *Cx. quinquefasciatus* and *Ma. africana* at the cluster level. This was compared between seasons (dry and wet) using a mixed effect generalized linear model with a negative binomial distribution. Collection rounds were included in the model as random effects. The seasons were included as a fixed effect. Chi-square test was used to analyse the proportions of sibling species identified in *An. gambiae* s.l. Stata 15.0 software (Stata Corp., College Station, TX, USA) was used for these analyses.

Ecological niche models were produced using MaxEnt version 3.4.4 software^31^. The maps were produced using ArcGIS version 10.8 software^34^.

### Ethics approval

The study protocol received ethical approval from both Benin's National Ethics Committee for Health Research (Reference N°30/MS/DC/SGM/DRFMT/CNERS/SA, Approval n°6 of 04/03/2019) and the Ethics Committee of the London School of Hygiene and Tropical Medicine (Approval 16237-1). All participants provided informed consent prior to their involvement. All collectors underwent mosquito capture training that will allow them to avoid bites during the collection. Health measures taken included yellow fever vaccination before the study and access to local healthcare facilities for treatment of confirmed malaria cases or other ailments with similar symptoms.

## Results

### Composition and relative abundance of mosquito species

**Figure 2:**
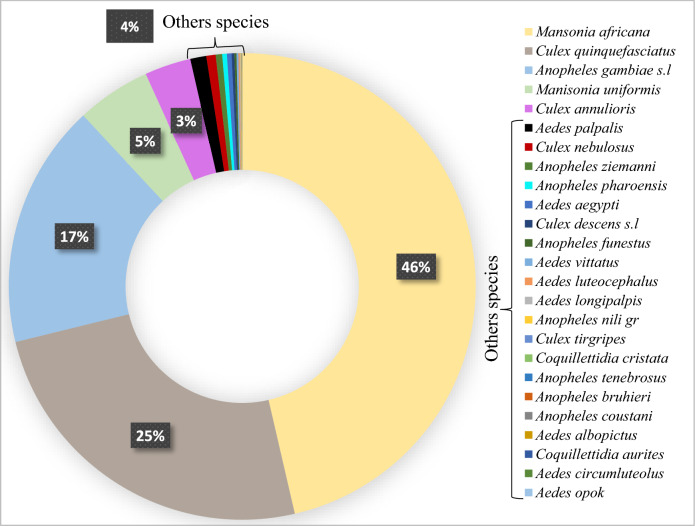
Composition and relative abundance of mosquito species.

A total of 161,582 mosquitoes were collected. Of these, Culicinae mosquitoes were the most abundant representing (82.1%, n = 132,662), followed by Anophelinae (17.9%, n = 28,920). Overall, collected mosquitoes were morphologically identified into 5 genera: *Aedes (Ae.)*, *Anopheles (An.)*, *Coquillettidia (Cq.)*, *Culex* (*Cx*.) and *Mansonia (Ma.)*, with a total of 25 different species (Fig. 2).

Overall, *Mansonia* spp. (52%, n = 83,241), comprised of 2 species, were the most abundant mosquitoes, followed by *Culex* spp. (29%, n = 46,641) and *Anopheles* spp. (18%, n = 28,920), which had 5 and 8 species, respectively. Nighttime HLCs collected few *Aedes* (1.7% of the catches, n = 2771), and *Coquillettidia* (< 0.1%, n = 9).

In *Mansonia* spp., *Ma. africana* was the most predominant, accounting for 90% (74,945 out of 83,241) of the total number of individuals of this genus. In *Culex* spp., *Cx. quinquefasciatus* and *Cx. annulioris* accounted for 85.9% (40,058 out of 46,641) and 11.1% (5185 out of 46,641) respectively. In *Anopheles* spp. the most frequent species mosquito species was *An. gambiae* s.l. (96.9%).

### Seasonal abundance and diversity of mosquito species

**Figure 3:**
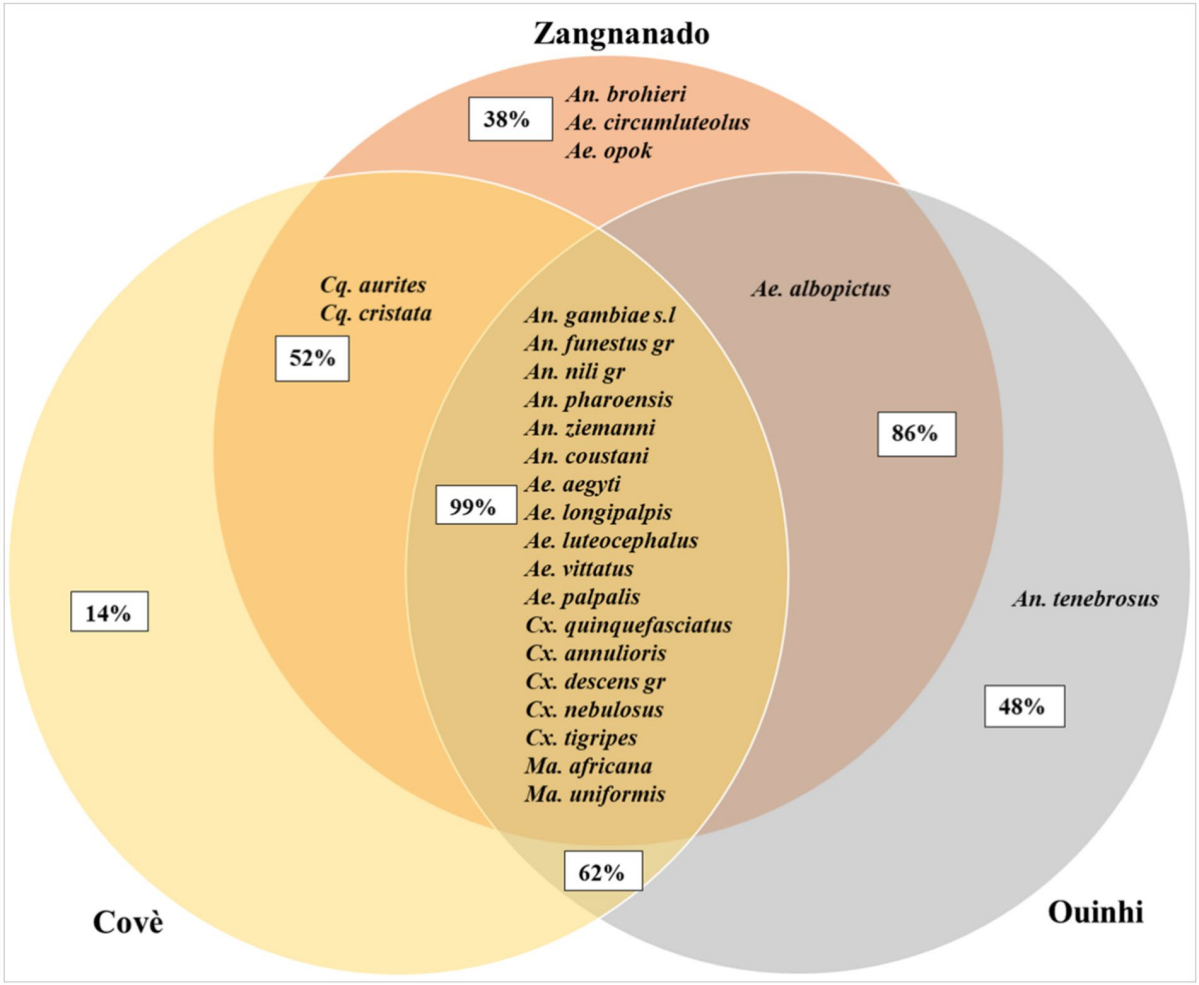
Venn diagram showing mosquito species collected at Covè (gold), Zangnanado (orange), and Ouinhi (grey). *An.*, *Anopheles*; *Ae.*, *Aedes*; *Cx.*, *Culex*; *Ma.*, *Mansonia*; *Cq*., *Coquillettidia.*

The species diversity within mosquito populations was assessed in the 3 communes. The highest species richness was observed in Zangnanado with 24 mosquito species collected, compared to 20 species for both Covè and Ouinhi (Fig. 3). A total of 18 mosquito species were common to all three study communes (Fig. 3). Overall, lower species richness was observed during the dry season compared to the wet season (21 vs 25 respectively).

Overall, there is no evidence for a higher density of *An. gambiae* s.l in the dry season compared to the wet season in Covè (31.1 bites/person/night (b/p/n) vs 22.2 b/p/n, p = 0.5984) and Ouinhi (14.7 b/p/n vs 14.1 b/p/n, p = 0.3445). By comparison, a significantly greater density of the same mosquito species was observed in the dry season (12.6 b/p/n) than in the wet season (9.8 b/p/n) in Zangnanado (p = 0.0001) (Table 1).

**Table 1:** Seasonal abundance of mosquito species per commune.

Localities	Seasons	Mean density per night (95%IC)			Indices			
		*An. gambiae *s.l.	*Cx. quinquefasciatus*	*Ma. africana*	Number of species (S)	Simpson (1−D)	Shannon (Hʹ)	Equitability (J)
Covè	Wet	22.2^a^ (9.6–34.8)	15.2^e^ (6.6–23.9)	64.9^k^ (35.7–94.2)	18	0.59^q^	1.18^t^	0.58^z^
	Dry	31.1^a^ (2.2–64.49)	7.4f. (2.0–12.7)	14.4^l^ (5.5–23.4)	16	0.51^q^	0.94^u^	0.57^z^
Zangnanado	Wet	9.8^b^ (7.2–12.3)	17.0^ g^ (9.0–24.9)	37.8^m^ (20.2–55.4)	23	0.52^r^	1.03^v^	0.57^e^
	Dry	12.6^c^ (6.0–19.3)	11.4^ h^ (4.6–18.2)	13.1^n^ (4.1–22.2)	20	0.50^r^	0.90^w^	0.69^f^
Ouinhi	Wet	14.1^d^ (7.3–20.9)	43.1^i^ (32.5–53.8)	109.8° (79.2–140.4)	20	0.55^s^	1.09^x^	0.50^z^
	Dry	14.7^d^ (6.1–23.4)	30.1^j^ (17.1–44.0)	15.0^p^ (10.6–19.4)	15	0.49^s^	0.90^y^	0.56^z^

Indicator values with different superscripts within the same commune for the same mosquito species are significantly different (p < 0.05). The mean density was expressed in terms of number of bites/person/night (b/p/n).

The density of *Cx. quinquefasciatus* was significantly lower in the dry season than in the wet season in Covè (7.4 b/p/n vs 15.2 b/p/n, p < 0.0001), Zangnanado (11.4 b/p/n vs 17.0 b/p/n, p < 0.0001) and Ouinhi (30.1 b/p/n vs 43.1 b/p/n, p < 0.0001) (Table 1). The same trend was observed for *Ma. africana* in Covè (14.4 b/p/n vs 64.9 b/p/n, p < 0.0001), Zangnanado (13.1 b/p/n vs 37.8 b/p/n, p < 0.0001) and Ouinhi (15.0 b/p/n vs 109.8 b/p/n, p < 0.0001) (Table 1).

The Shannon–Weaver index assessing mosquito diversity was 0.90–1.18, with the highest value recorded in Covè. Analysis of this index indicated a significant difference in mosquito population diversity between wet and dry seasons in each of the three study communes (p < 0.05) (Table 1).

The Piélou equitability index ranged between 0.50 (Ouinhi) and 0.69 (Zangnanado) and was similar (p > 0.05) between dry and wet seasons in Covè and Ouinhi. However, in Zangnanado, the Piélou equitability index was significantly higher in the dry season as compared to the wet season (p = 0.0061) (Table 1).

The Simpson index varied between 0.49 (Ouinhi) and 0.59 (Covè), and was similar (p > 0.05) between the wet and dry seasons in each of the three communes (Table 1).

Irrespective of the season, *An. coluzzii* and *An. gambiae* s.s. were the two sibling species identified in the *An. gambiae* complex in the three study communes (Table 2).

**Table 2:** Seasonal abundance of *Anopheles gambiae* complex species per commune per season.

Locality/season	Total assayed	*An. coluzzi*	*An. gambiae* s.s.	*An. gambiae* s.s.*/coluzzii*
		% (95% CI), N	% (95% CI), N	% (95% CI), N
Overall				
Covè	772	70.2 (66.8–73.4), 542	29.5 (26.4–32.9), 228	0.3 (0.0–1.0), 2
Zangnanado	1854	52.9 (50.5–55.1), 980	47.0 (44.7–49.3), 871	0.2 (0.0–0.5), 3
Ouinhi	1354	94.4 (93.0–95.5), 1278	5.2 (4.1–6.6), 71	0.4 (0.1–0.9), 5
Wet season				
Covè	498	64.5 (60.1–68.6), 321	35.5 (31.4–39.9), 177	0.0 (0.0–0.9), 0
Zangnanado	1163	45.4 (42.5–48.3), 528	54.4 (51.5–57.3), 633	0.2 (0.0–0.7), 2
Ouinhi	803	94.0 (92.1–95.5), 755	5.6 (4.2–7.5), 45	0.4 (0.1–1.2), 3
Dry season				
Covè	274	80.7 (75.4–85.1), 221	18.6 (14.3–23.8), 51	0.7 (0.1–02.9), 2
Zangnanado	691	65.4 (61.7–68.9), 452	34.4 (30.9–38.1), 238	0.1 (0.0–0.9), 1
Ouinhi	551	94.9 (92.6–96.5), 523	4.7 (3.2–6.9), 26	0.36 (0.1–1.4), 2

*An*, *Anopheles*; N, number of; %, proportion; CI, confidence interval.

All seasons (wet and dry) combined, *An. coluzzii* was the most abundant mosquito species, accounting for 70.2% (66.8–73.4), 52.9% (50.5–55.1), and 94.4% (93.0–95.5) of all mosquitoes sampled in Cove, Zangnanado, and Ouinhi, respectively. A similar trend was observed during both dry and wet seasons (Table 2).

Hybrid specimens (*An. gambiae* s.s*./coluzzii*) were collected in very low proportions (< 1%) in all three communes during both seasons (Table 2).

### Ecological niche models for mosquito species

**Figure 4:**
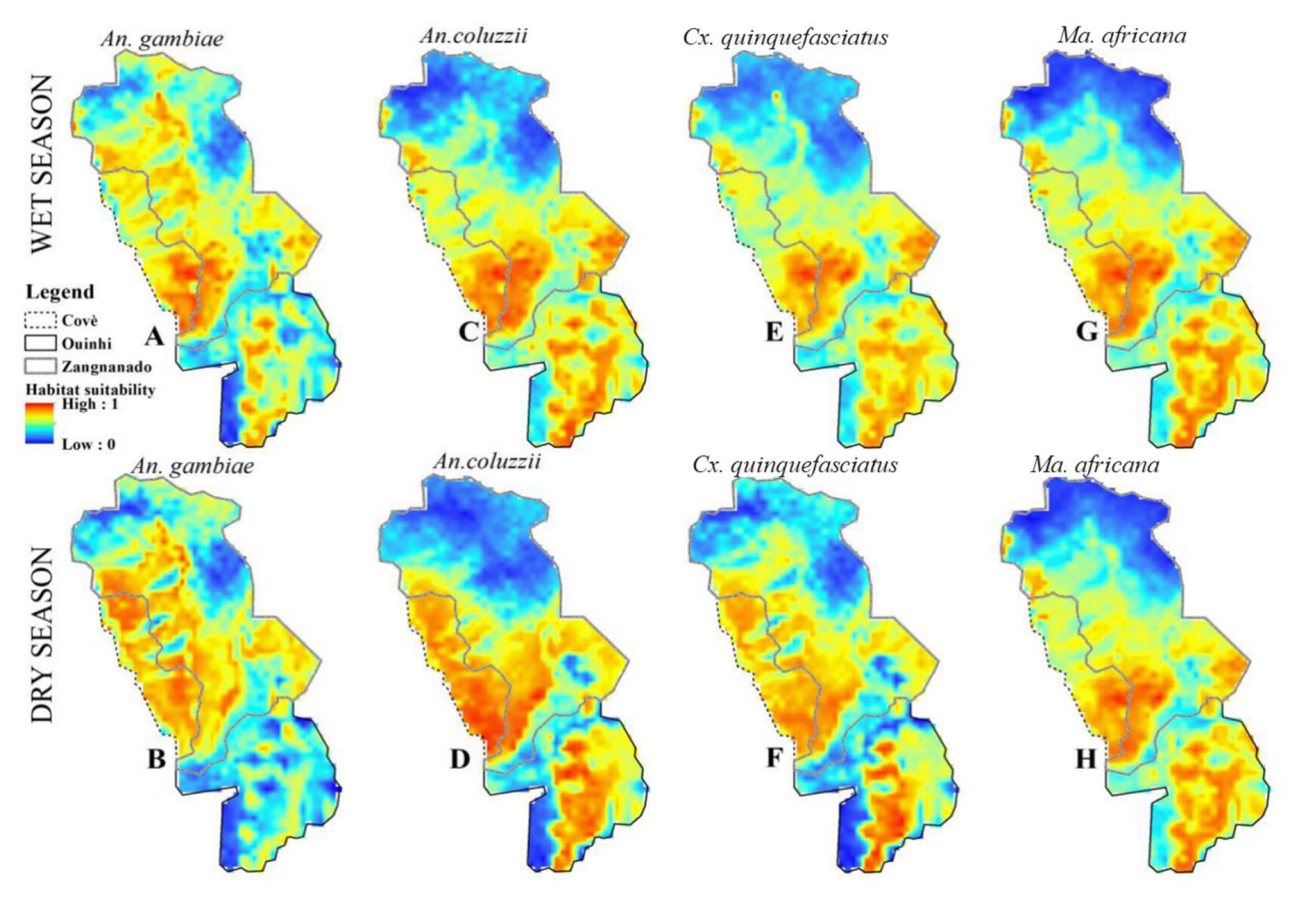
Habitat suitability models in wet and dry seasons for *An. gambiae* s.s. (**A** and **B**), *An. coluzzii* (**C** and **D**), *Cx. quinquefasciatus* (**E** and **F**) and *Ma. africana* (**G** and **H**) under current climatic conditions.

During the wet season, *An. gambiae* s.s., *An. coluzzii*, *Cx. quinquefasciatus*, and *Ma. africana* showed very high habitat suitability in the south of Covè, south-west and east of Zangnanado, and centre of Ouinhi (Fig. 4). However, the north of Covè and north-western of Zangnanado showed moderate to high suitability for *An. gambiae* s.s. (Fig. 4A). The models also revealed high habitat suitability in the north of Zangnanado and south-west of Ouinhi during the same period for *An. coluzzii, Cx. quinquefasciatus* and *Ma. africana* (Fig. 4C,E,G, respectively).

In the dry season, very high habitat suitability was observed throughout the commune of Covè for *An. gambiae* s.s., *An. coluzzii* and *Cx. quinquefasciatus* (Fig. 4B,D,F). The centre of Ouinhi was a highly favourable habitat for *An. coluzzii, Cx. quinquefasciatus* and *Ma. africana* (Fig. 4D,F,H). The west and south-east of Zangnanado showed high habitat suitability for all four mosquito species (Fig. 4B,D,F,H).

### Contribution of variables to mosquito habitat suitability in the Covè-Zangnanado-Ouinhi health zone

**Figure 5:**
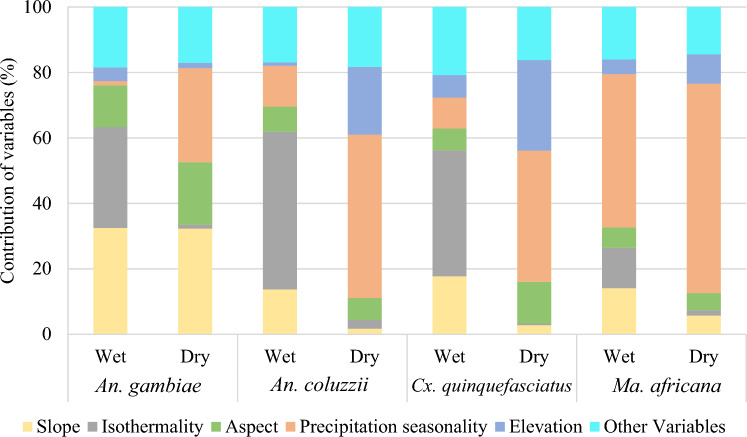
Contribution of variables in mosquito species models during the wet and dry seasons.

Figure 5 shows the impact of the different evaluated bioclimatic and environmental data on the habitat suitability of *An. gambiae* s.s, *An. coluzzii, Cx. quinquefasciatus* and *Ma. africana*.

For *An. gambiae* s.s, slope and isothermality were the most important predictors of habitat suitability (Fig. 5). Thus, habitat suitability for this mosquito species increases when slope decreases (wet and dry seasons), and isothermality increases (wet season).

Regarding *An. coluzzii*, habitat suitability was positively correlated with isothermality (wet season), and negatively correlated with precipitation seasonality (dry season).

For *Cx. quinquefasciatus*, the habitat was most suitable when the isothermality increased (wet season), and the precipitation seasonality decreased (dry season).

*Ma. africana* habitat suitability increased as the precipitation seasonality decreased in both wet and dry seasons (Fig. 5).

## Discussion

The present study focussed on the diversity, spatio-temporal distribution and ecological preferences of mosquito populations from the communes of Covè, Zangnanado and Ouinhi, areas endemic for malaria and lymphatic filariasis^16,35^. Overall, all studied mosquito species were more abundant in the wet season, except for *An. gambiae* s.l. whose density was higher in the dry season. Regardless of the commune, *An. coluzzii* predominated over *An. gambiae* s.s. in all seasons (wet and dry). Mosquito species diversity was higher in the wet season. The habitat suitability of *An. gambiae* s.s., *An. coluzzii*, *Ma. africana* and *Cx. quinquefasciatus* were mainly dependent on slope and precipitations, with some variations between species and seasons.

Overall, a total of 25 mosquito species belonging to five different genera were collected. The Culicinae composed mainly of *Mansonia* spp., and *Culex* spp., were significantly more abundant than the Anophelinae made up largely of *An. gambiae* s.l. This trend is similar to that observed in several entomological monitoring trials carried out both in Benin^36,37^ and in other countries^38,39^. Mosquitoes of the *Aedes* genus were collected in low proportions, which could be due to the collection methods used, as well as the diurnal activity of this mosquito genus. Indeed, previous studies have shown that the collection of *Aedes* mosquitoes was greater with BG Sentinel Traps^40–42^, with peaks of activity usually observed early in the morning and in the afternoon^43^.

Our results showed a seasonal variation in the density and diversity of mosquito species in each study commune. The high densities of *Cx. quinquefasciatus* and *Ma. africana* during the wet season, a period conducive to the development of mosquito breeding sites, are consistent with previous observations made in India, Kenya, and Cambodia^44–46^. The unusual observation of *An. gambiae* s.l. that revealed to be more abundant in the dry season, could be because of the presence of flowing rivers in the low altitude areas of the study communes. These rivers are suitable for irrigated crops such as rice growing and market gardening, which create suitable habitats for the development of larvae of *An. gambiae* s.l.^47^ In addition, the withdrawal of the Ouémé and Zou rivers from their beds during the dry season created numerous breeding sites favourable to this mosquito species. A similar observation was made around the Sanaga River in Southern Cameroon^48^.

The diversity of mosquito populations is a paramount indicator as it provides information on the risk of mosquito-borne diseases^49^. In the three study communes, this index was higher in the wet season than in the dry season. This suggests that the mosquito populations of the study communes had few dominant species. Indeed, *Ma. africana, Cx. quinquefasciatus* and *An. gambiae s.l.* were the three most abundant mosquito species as they accounted for 85% (98,169/115,550) of the total number of mosquitoes sampled during the wet season. This suggests that the breeding sites in these communes were more suitable for the proliferation of these three mosquito species. This high diversity could increase the risk of transmission of mosquito-borne diseases such as malaria, and lymphatic filariasis^49^.

The study of ecological niche patterns revealed crucial information about the seasonal distribution of mosquito species. During the wet season, *An. gambiae* s.s.*, An. coluzzii, Cx. quinquefasciatus* and *Ma. africana* were mainly present in the south of Covè, the southwest and east of Zangnanado, as well as the centre of Ouinhi. This shows that these species share a similar ecological niche in the wet season. Indeed, these regions are low-altitude floodplains where water and vegetation coexist, thus forming semi-permanent breeding sites favourable to the development of *An. coluzzii*^50^, *Cx. quinquefasciatus* and *Ma. africana*. This concurs with the higher proportion of *An. coluzzii* compared to *An. gambiae* s.s. recorded in the area. The colonisation of *An. gambiae* s.s. in the north of Covè, and the northwest of Zangnanado (high altitude areas) during this period, could be due to the numerous temporary breeding sites created by rainwater, as previously reported by Minakawa et al*.*^51^ in Kenya.

During the dry season, the entire commune of Covè, the centre of Ouinhi, as well as the west and southeast of Zangnanado (areas consisted of plains previously flooded and where the recession started) offered highly suitable habitats for all four species of mosquitoes due to the recession of the flood. The same observation was made around the Gambia^52^ and Ghibe^53^ rivers. From the wet season to the dry season, the observation made is that suitable habitats for mosquitoes seem to move from the northeast to the southwest. This could be due to the direction of the flow of the Ouémé River which is influenced by the slope.

According to the work of Accrombessi et al.^54^ conducted in the study area, sites with a high density of *An. gambiae* s.l. were associated with a high prevalence of malaria, so it would be judicious to focus malaria vector control interventions in areas where ecological niche models revealed suitable habitats for this mosquito species.

Past studies have highlighted several environmental factors that influenced the distribution of different mosquito species^55,56^. In the present study, our models revealed that precipitation seasonality, slope, isothermality and elevation were particularly relevant variables in the distribution of *An. gambiae* s.s., *An. coluzzii*, *Cx. quinquefasciatus* and *Ma. africana*. Indeed, the slope was important in determining in the distribution of *An. gambiae* s.s., while the elevation significantly influenced the distribution of *An. coluzzii*, *Cx. quinquefasciatus*, and *Ma. africana*. These results corroborate those obtained with *Cx. quinquefasciatus* in the Galápagos^57^.

Although the present study provides useful information on the abundance and diversity of mosquito species, as well as the ecological factors that influence their distribution, it has some limitations such as: the lack of consideration of socio-economic factors (demography, level of urbanization, agricultural activity, etc.), and the availability of hosts in the analyses; the difficulty in generalizing the results obtained to other regions due to the variability in the ecology, the short study period (one year); the failure to take into account complex interactions between species (predation, parasitism, competition) in the calculation of diversity indices; and the reliance on one mosquito collection technique. Another major gap for the present study is also the lack of *Plasmodium* sporozoite infection data.

## Conclusion

The present study is a significant contribution to the understanding of the ecology of the main mosquito species encountered in the communes of Covè, Ouinhi and Zangnanado in Southern Benin. Indeed, it shows that the abundance and diversity of these mosquito species were much more pronounced in the wet season than in the dry season, with ecological preferences varying depending on the season. These findings will enable the implementation of targeted control strategies that take into account both the ecological preferences of each mosquito species as well as their seasonality.

### Supplementary Information

Supplementary Table S1.

## Data Availability

Datasets analysed as part of this study are available from the corresponding author upon reasonable request.
