# Supplementary material for: Diversity and ecological niche model of malaria vector and non-vector mosquito species in Covè, Ouinhi, and Zangnanado, Southern Benin
Source: Sci Rep. 2024 Jul 23;14:16944. doi: 10.1038/s41598-024-67919-5 (PMC11266568; doi:10.1038/s41598-024-67919-5)
Supplement: Supplementary file 1 — Supplementary Table S1. [file 41598_2024_67919_MOESM1_ESM.docx]

# **Supplementary material**

**Table S1.** Climatic and environmental layers name and processing in the Model

| **Layer name** | **Processing** | **References** |
| --- | --- | --- |
| **Climate** |  |  |
| Bio1: Annual mean temperature | Clipped raster at 1000m resolution | ^48^ |
| Bio2: Mean diurnal range | Clipped raster at 1000m resolution | ^48^ |
| Bio3: Isothermality | Clipped raster at 1000m resolution | ^48^ |
| Bio4: Temperature seasonality | Clipped raster at 1000m resolution | ^48^ |
| Bio5: Maximum temperature of warmest period | Clipped raster at 1000m resolution | ^48^ |
| Bio6: Minimum temperature of coldest period | Clipped raster at 1000m resolution | ^48^ |
| Bio7: Temperature annual range | Clipped raster at 1000m resolution | ^48^ |
| Bio8: Mean temperature of wettest quarter | Clipped raster at 1000m resolution | ^48^ |
| Bio9: Mean temperature of driest quarter | Clipped raster at 1000m resolution | ^48^ |
| Bio10: Mean temperature of warmest quarter | Clipped raster at 1000m resolution | ^48^ |
| Bio11: Mean temperature of coldest quarter | Clipped raster at 1000m resolution | ^48^ |
| Bio12: Annual precipitation | Clipped raster at 1000m resolution | ^48^ |
| Bio13: Precipitation of wettest month | Clipped raster at 1000m resolution | ^48^ |
| Bio14: Precipitation of driest month | Clipped raster at 1000m resolution | ^48^ |
| Bio15: Precipitation seasonality | Clipped raster at 1000m resolution | ^48^ |
| Bio16: Precipitation of wettest quarter | Clipped raster at 1000m resolution | ^48^ |
| Bio17: Precipitation of driest quarter | Clipped raster at 1000m resolution | ^48^ |
| Bio18: Precipitation of warmest quarter | Clipped raster at 1000m resolution | ^48^ |
| Bio19: Precipitation of coldest quarter | Clipped raster at 1000m resolution | ^48^ |
| **Environmental** |  |  |
| Aspect: Compass direction | Clipped raster at 1000m resolution | ^48^ |
| Elevation: Altitude | Clipped raster at 1000m resolution | ^48^ |
| Soil: Type of soil | Clipped raster at 1000m resolution | ^48^ |
| Slope: Steepness | Clipped raster at 1000m resolution | ^48^ |
| NDVI: Normalized Differential Vegetation Index | Clipped and resampled raster at 1000m resolution | ^53^ |
